# Increased Efficacy of Zinc Complexes With Picolinic and Aspartic Acids Against Herpes Simplex Virus (HSV) Infection When Combined With the Pavine Alkaloid (-)-Thalimonine

**DOI:** 10.1155/MBD.2000.257

**Published:** 2000

**Authors:** Assia L. Angelova, Tatiana L. Varadinova

**Affiliations:** University of Sofia Faculty of Biology Laboratory of Virology Dragan Tzankov Blvd 8 Sofia 1421 Bulgaria

## Abstract

Complexes of zinc with picolinic and aspartic acids inhibit key steps of HSV-1 replication affecting different virus-specific targets. As was recently demonstrated by us, the pavine alkaloid (-)-thalimonine irreversibly inhibits HSV-1 infection in cultured cells. The aim of the present study was the evaluation of the combined effect of zinc complexes and (-)-thalimonine on uninfected and HSV-1 infected cells. The data obtained have shown that zinc complexes and the alkaloid exert decreased cytotoxicity (antagonistic effect) and significantly increased anti-HSV-1 activity (synergistic effect) when applied in dual chess-board combinations as compared to the individual effects of compounds tested. These combinations are also effective against the infection caused by a resistant to acyclovir (ACV) HSV-1 mutant and the effect has been recognised as synergistic.


### INCREASED EFFICACY OF ZINC COMPLEXES WITH PICOLINIC AND ASPARTIC ACIDS AGAINST HERPES SIMPLEX VIRUS (HSV) INFECTION WHEN COMBINED WITH THE PAVINE ALKALOID (-)-THALIMONINE

Assia L. Angelova and Tatiana L. Varadinova*

University of Sofia, Faculty of Biology, Laboratory of Virology,Dragan Tzankov Blvd 8, 1421 Sofia
<tvar@ns.biofac.uni-sofia.bg>

**Abstract**

Complexes of zinc with picolinic and aspartic acids inhibit key steps of HSV-1 replication affecting different virus-specific targets. As was recently demonstrated by us, the pavine alkaloid (-)-thalimonine irreversibly inhibits HSV-1 infection in cultured cells. The aim of the present study was the evaluation of the combined effect of zinc complexes and (-)-thalimonine on uninfected and HSV-1 infected cells. The data obtained have shown that zinc complexes and the alkaloid exert decreased cytotoxicity (antagonistic effect) and significantly increased anti-HSV-1 activity (synergistic effect) when applied in dual chess-board combinations as compared to the individual effects of compounds tested. These combinations are also effective against the infection caused by a resistant to acyclovir (ACV) HSV-1 mutant and the effect has been recognised as synergistic.

**Introduction**

Our previous data have shown that complexes of zinc with picolinic and aspartic acids - $Zn(pic)_2$ and $Zn(asp)_2$, inhibit key steps of HSV-1 replication altering: ( i) the synthesis of essential viral regulatory proteins (ICP4, ICP8) and of structural glycoproteins (gD, gH) and, (ii) the morphogenesis of infectious viral progeny (1, 2).

Furthermore, the pavine alkaloid (-)-thalimonine, isolated from *Thalictrum simplex* (L.), negatively affects HSV-1 infection in cultured cells as was already demonstrated by us (3).

However, it is well known that during the systemic administration of antivirals, resistant HSV mutants appear with high frequency (4, 5). This problem could be solved by combination therapy using compounds which affect different virus-specific targets. That is why we decided to investigate whether it is possible to increase the effect of zinc complexes against HSV-1 infection when applied in combination with the alkaloid. A special attention was paid on the effectiveness of the combinations against the infection caused by a resistant to ACV HSV-1 mutant.

**Materials and Methods**

1. *Cells and viruses.* Continuous Madine - Darbey bovine kidney (MDBK) cells were grown in RPMI 1640 medium (GIBCO BRL) supplemented with 10% bovine serum (BS) and antibiotics. The same medium supplemented with 5% BS and antibiotics was used in the antiviral experiments. Two HSV-1 strains were used: the first one, Victoria, is sensitive to ACV, while the second one, R-100 ($ACV^R$, $TK^A$), is resistant due to a mutation in the viral gene encoding the enzyme thymidine kinase (TK). Viral stocks were prepared by infecting MDBK cells, harvesting at a full cytopathic effect (CPE), freezing and thawing three times and then stored at -70$^0$C.

2. *Compounds tested.* Zinc complexes with picolinic and with aspartic acids - $Zn(pic)_2$ and $Zn(asp)_2$, and the pavine alkaloid (-)-3,4-methylenedioxy-2,8,9-trimethoxypavinane, further designated as (-)-thalimonine, isolated from the aerial parts of the mongolian plant *Thalictrum simplex* (L.), Ranunculaceae, were used in the combinatory experiments.

3. *Single-cycle growth experiments.* MDBK cells from confluent monolayers were infected with HSV-1, strain Victoria. After 1h for attachment cells were covered with media modified with 10 μM of the appropriate compound. At the 2h, 4h, 8h, 10h, 12h and 15h samples were freezed, thawed and titrated in 96-well microplates.The data were compared with the viral controls - infected cells cultured in unmodified medium and collected at the same intervals. The infectiuos viral titres were expressed as $\log_{10}$ of infectious units (i.u.) per 0.1ml and as a per cent from the viral control.

4. *Combinatory assays.*

4.1. *Cytotoxicity assay.* Confluent monolayers from MBDK cells grown in 96-well plates were covered with medium supplemented with compounds tested, either alone or in dual combination. The cytotoxicity was evaluated on the 48h, 72h and 96h after culturing cells at 37$^0$C by trypan blue exclusion test. Cells covered with unmodified, compound-free medium and cultured in the same experimental conditions served as a control. The concentration which reduced the cell survival rate with 50% as compared to the untreated control was designated as 50% cytotoxic ($CC_{50}$).

4.2. *Antiviral* assay. Confluent monolayers were infected with the appropriate HSV-1 strain in ten-fold dilutions. After 1h for adsorption cells were covered with medium modified with the compounds tested, either alone or in dual combinations. The infectious viral titres were determined on the 48h or 72h after infection with strain Victoria or R-100, respectively. The anti-HSV-1 activity was expressed as a reduction of the viral titres as compared to the control - infected cells covered with unmodified medium. The concentration required to reduce the infectious viral titre with 50% was designated as 50% inhibitory ($IC_{50}$).
Data from the combinatory assays were estimated by the three-dimensional model of Prichard and Shipman (6). According to this model, synergistic or antagonistic effects can be identified using dual chess-board combinations of compounds applied in the following concentrations: $2CC_{50}$, $CC_{50}$, $CC_{50}/2$, $CC_{50}/4$, $CC_{50}/8$ or: $2IC_{50}$, $IC_{50}$, $IC_{50}/2$, $IC_{50}/4$ and $IC_{50}/8$. Based on the experimentally observed results from toxicity and antiviral experiments, the theoretical additive effect was calculated and subtracted from the observed effect. The data after subtraction were presented graphically where values below 0 were indicative for antagonostic effect, while those above 0 - for synergistic effect.

**Results and discussion**

Single-cycle growth tests are useful for characterisation of viral replication specificities. These tests are also informative when the target period during which the particular compound specifically suppresses viral replication, has to be identified. As is shown in fig. 1, the compounds tested affect HSV-1 infection in a different manner when evaluated in single-cycle growth conditions.

Fig. 1 Effect of $Zn(pic)_2$, $Zn(asp)_2$ and (-)-thalimonine on HSV-1 infection in single-cycle growth conditions

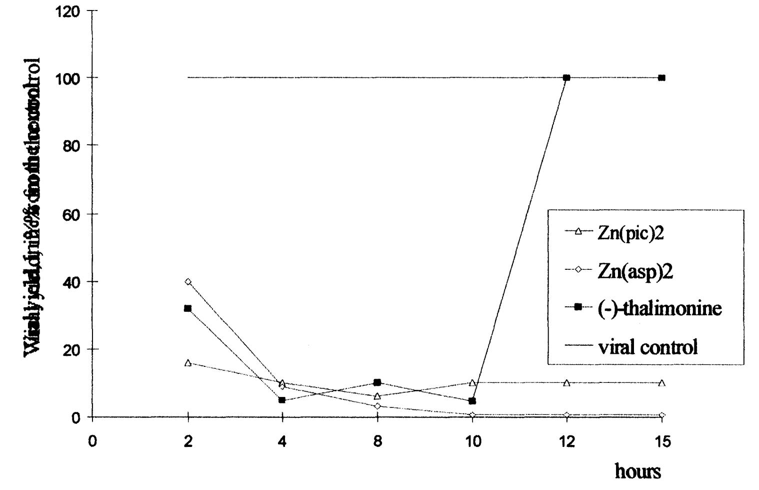

Thus, $Zn(pic)_2$ and $Zn(asp)_2$ negatively affect the whole period of HSV-1 replication suppressing the expression of immediate early (IE), early (E) and late (L) gene products. These results confirm the data previously reported by us (1, 2). Using monoclonal antibodies, immunoblotting and electron microscopy it was shown that $Zn(pic)_2$ and $Zn(asp)_2$ inhibit essential regulatory and structural HSV-1 proteins (1, 2) and the morphogenesis of viral progeny (1). However, the peak of activity of $Zn(pic)_2$ has been measured on the 8h post infection, respectively 7h after the treatment and, at the end of the replication cycle 10% of infectious viral progeny is produced. As concern $Zn(asp)_2$, its suppressive activity progressively increases with the prolongation of action and on the 15h 0.6% of infectious viral progeny is only produced. Contrary, the alkaloid (-)-thalimonine affects the early period of HSV-1 replication, as the amount of infectious viral progeny is reduced between the 4h and 10h after the infection, respectively 3h - 9h after the treatment. Till the end of the replicative cycle (-)-thalimonine is uneffective, as demonstrated by the restored viral infectivity on the 12h and 15h, reaching control level (fig. 1).
The fact that zinc complexes and (-)-thalimonine affect different periods of HSV-1 replication motivated us to use these compounds in dual combinatory experiments.
In the first set of experiments individual $CC_{50}$ values of compounds tested were evaluated. The respective $CC_{50}$ are 100 μM for both zinc complexes and 30 μM for (-)-thalimonine. In the next set of experiments, based on the individual $CC_{50}$, the cytotoxicity of combinations was evaluated (tables 1 and 2, fig. 2 and 3).

Table 1. Observed cytotoxic effect of $Zn(pic)_2$ and (-)-thalimonine applied alone and in combination *

| | (-)-thalimonine | | | | | |
|---|---|---|---|---|---|---|
| **$Zn(pic)_2$** | 0 | $CC_{50}/8$ | $CC_{50}/4$ | $CC_{50}/2$ | $CC_{50}$ ♦ | $2CC_{50}$ |
| $2CC_{50}$ | 25 | 23.5 | 21 | 18 | 14.5 | 11 |
| $CC_{50}$ ° | 50 | 35 | 30 | 24.5 | 19 | 15 |
| $CC_{50}/2$ | 60 | 55 | 37 | 28 | 20 | 18.5 |
| $CC_{50}/4$ | 71 | 60 | 51 | 35 | 30 | 25 |
| $CC_{50}/8$ | 75 | 70 | 62 | 55.5 | 45 | 30 |
| 0 | 100 • | 77 | 75 | 62 | 50 | 35 |

* data shown in per cent of the cell control; • cell control; ° 100 μM; ♦ 30 μM

Table 2. Observed cytotoxic effect of $Zn(asp)_2$ and (-)-thalimonine applied alone and in combination *

| | (-)-thalimonine | | | | | |
|---|---|---|---|---|---|---|
| **$Zn(asp)_2$** | 0 | $CC_{50}/8$ | $CC_{50}/4$ | $CC_{50}/2$ | $CC_{50}$ ♦ | $2CC_{50}$ |
| $2CC_{50}$ | 30 | 25 | 20 | 18 | 15.5 | 10 |
| $CC_{50}$ ° | 50 | 30 | 25.5 | 15.5 | 11 | 10 |
| $CC_{50}/2$ | 60 | 51.5 | 48 | 32 | 27.5 | 20 |
| $CC_{50}/4$ | 75 | 62 | 58.5 | 53 | 46 | 35 |
| $CC_{50}/8$ | 82 | 71 | 66.6 | 59.5 | 49 | 37 |
| 0 | 100 • | 75 | 70 | 63 | 50 | 38 |

* data shown in per cent of the cell control; • cell control; ° 100 μM; ♦ 30 μM

Fig. 2 Combined cytotoxic effect of Zn(pic)2 and (-)-thalimonine

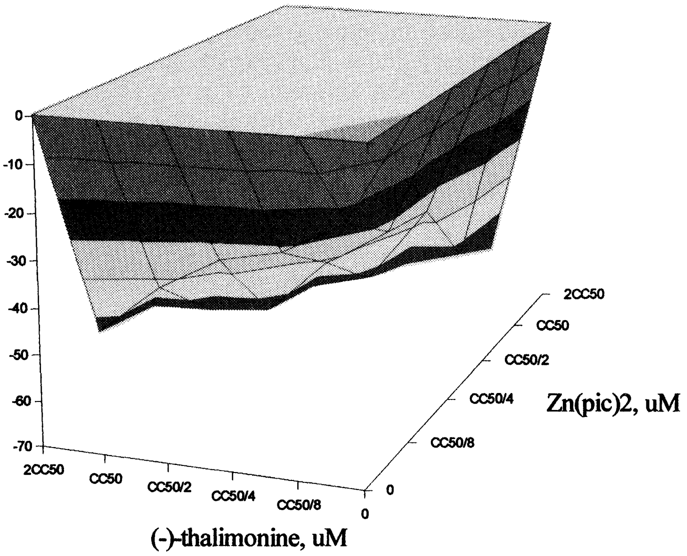

The data demonstrate that when the appropriate zinc complex is simultaneously applied with (-)-thalimonine, the observed cytotoxic effect is antagonistic, i.e. the compounds neutralize their cytotoxicity when used in combination. This is manifested by the decreased cytotoxicity of combinations as compared to that of compounds applied alone. Thus, the cytotoxicity of the combination $Zn(pic)_2$ and (-)-thalimonine is up to 54% lower (table 1, fig. 2), while that of combination $Zn(asp)_2$ and (-)-thalimonine is up to 50% lower than that obtained under the single action of compounds tested (table 2. fig. 3).

Fig. 3 Combined cytotoxic effect of Zn(asp) 2 and (-)-thalimonine

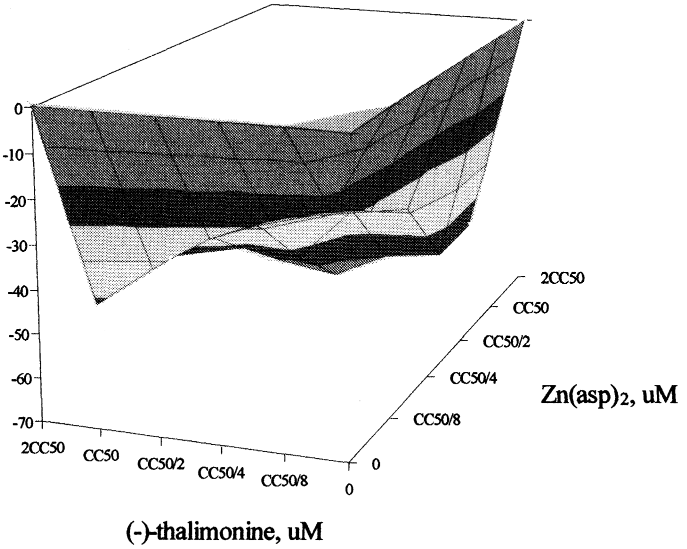

The antagonistic cytotoxic effect expressed by combinations used encouraged us to continue our experiments in order to identify the dual effect of zinc complexes and (-)-thalimonine on HSV-1 infection in the same cell culture system. The antiviral efficacy of combinations was compared to that after single application of the particular compound.

The experimental data on the combined effect of $Zn(pic)_2$ and (-)-thalimonine against strain Victoria show that the inhibitory effect is increased as compared to that of $Zn(pic)_2$ and (-)-thalimonine applied alone (table 3, fig. 4). According to the method of Prichard and Shipman, the antiviral effect of the combination can be defined as synergistic. The synergism is most pronounced when $IC_{50}/2$ $Zn(pic)_2$ is combined with $IC_{50}/2$ (-)-thalimonine (fig. 4).

Fig. 4 Combined effect of Zn(pic) 2 and (-)-thalimonine on the replication of HSV-1strain Victoria

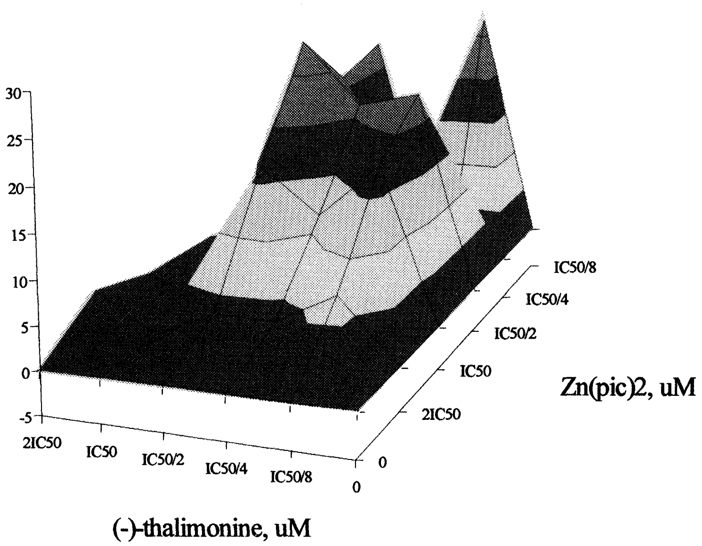

Table 3. Combined effect of $Zn(pic)_2$ and (-)-thalimonine on the replication of HSV-1 strain Victoria *

| | (-)-thalimonine | | | | | |
|---|---|---|---|---|---|---|
| **$Zn(pic)_2$** | 0 | $IC_{50}/8$ | $IC_{50}/4$ | $IC_{50}/2$ | $IC_{50}$ ♦ | $2IC_{50}$ |
| $2IC_{50}$ | 68 | 75 | 80 | 83.4 | 90 | 96.5 |
| $IC_{50}$° | 50 | 75 | 80 | 83.4 | 83.4 | 90 |
| $IC_{50}/2$ | 27 | 50 | 68 | 75 | 80 | 80 |
| $IC_{50}/4$ | 27 | 27 | 50 | 68 | 75 | 80 |
| $IC_{50}/8$ | 0 | 27 | 27 | 50 | 68 | 75 |
| 0 | 100 • | 0 | 27 | 27 | 68 | 75 |

• data shown in per cent inhibition as compared to the viral control; • viral control; infectious viral titre 4.33 $\log_{10}$ i.u./0.1 ml; ° 10 μM; ♦ 0.001 μM

The effect of combination of $Zn(asp)_2$ and (-)-thalimonine against the infection caused by strain Victoria is also synergistic (table 4, fig. 5). The synergism is most pronounced when $IC_{50}/4$ $Zn(asp)_2$ is combined with $IC_{50}/2$ of (-)-thalimonine (fig. 5).

Fig.5 Combined effect of $Zn(asp)_2$ and (-)-thalimonine on the replication of HSV-1strain Victoria

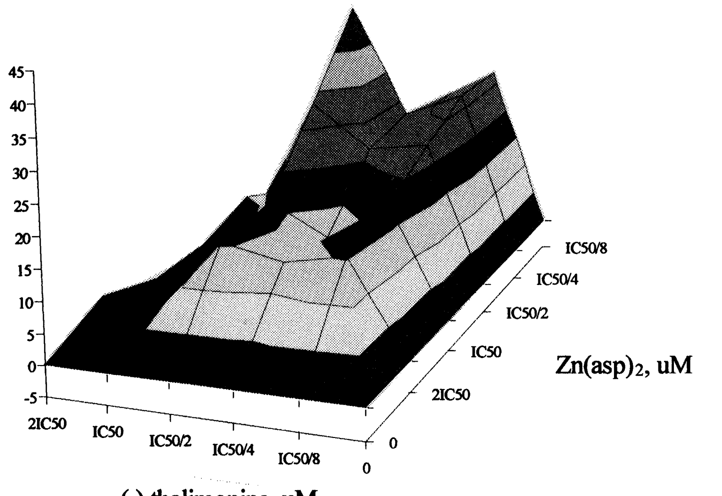

Table 4. Combined effect of $Zn(asp)_2$ and (-)-thalimonine on the replication of HSV-1 strain Victoria *

| | (-)-thalimonine | | | | | |
|---|---|---|---|---|---|---|
| **$Zn(asp)_2$** | 0 | $IC_{50}/8$ | $IC_{50}/4$ | $IC_{50}/2$ | $IC_{50}$ ♦ | $2IC_{50}$ |
| $2IC_{50}$ | 68 | 75 | 80 | 83.4 | 88.9 | 96.5 |
| $IC_{50}$ ° | 50 | 68 | 75 | 80 | 83.4 | 94 |
| $IC_{50}/2$ | 27 | 50 | 68 | 75 | 80 | 92 |
| $IC_{50}/4$ | 0 | 27 | 50 | 68 | 75 | 90 |
| $IC_{50}/8$ | 0 | 27 | 27 | 50 | 68 | 83.4 |
| 0 | 100 • | 0 | 27 | 27 | 68 | 75 |

* data shown in per cent inhibition as compared to the viral control; • viral control; infectious viral titre 4.33 $\log_{10}$ i.u./0.1ml; ° 25 μM; ♦ 0.001 μM

The experimental data showing the synergistic effect of combinations against the infection caused by the resistant to ACV strain R-100 represent significant interest (tables 5 and 6). The combination of $IC_{50}/4$ (-)-thalimonine and $IC_{50}/2$ $Zn(pic)_2$ or $Zn(asp)_2$ is the most effective (fig. 6 and 7). However, it has to be noted that when applied in low concentrations, such as $IC_{50}/8$, the combination of zinc complexes and (-)-thalimonine still exert a significant synergistic activity against ACV resistant strain R-100.

Table 5. Combined effect of $Zn(pic)_2$ and (-)-thalimonine on the replication of HSV-1 strain R-100 ($ACV^R$, $TK^A$) *

| | (-)-thalimonine | | | | | |
|---|---|---|---|---|---|---|
| **$Zn(pic)_2$** | 0 | $IC_{50}/8$ | $IC_{50}/4$ | $IC_{50}/2$ | $IC_{50}$ ♦ | $2IC_{50}$ |
| $2IC_{50}$ | 68 | 68 | 80 | 83.4 | 90 | 94 |
| $IC_{50}$ ° | 50 | 68 | 68 | 75 | 80 | 83.4 |
| $IC_{50}/2$ | 27 | 50 | 50 | 68 | 75 | 83.4 |
| $IC_{50}/4$ | 27 | 27 | 50 | 68 | 68 | 75 |
| $IC_{50}/8$ | 0 | 27 | 27 | 50 | 68 | 75 |
| 0 | 100 • | 0 | 0 | 27 | 50 | 68 |

* data shown in per cent inhibition as compared to the viral control; • viral control; infectious viral titre 4.3 $log_{10}$ i.u./0.1ml; ° 20 μM; ♦ 0.0000001 μM

Fig. 6 Combined effect of $Zn(pic)_2$ and (-)-thalimonine on the replication HSV-1strain R-100

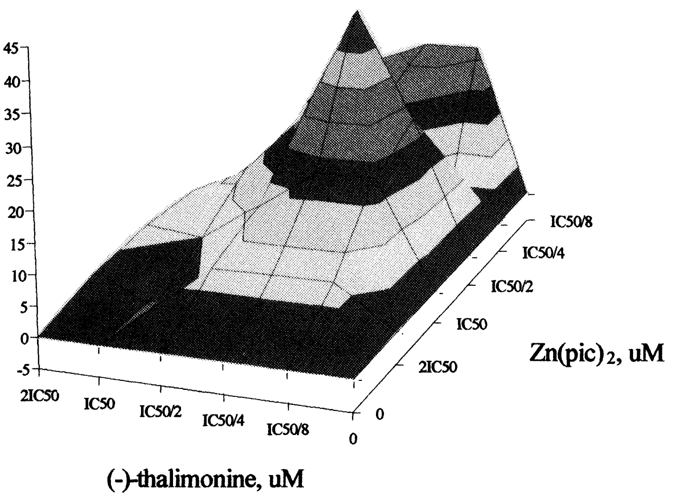

Table 6. Combined effect of $Zn(asp)_2$ and (-)-thalimonine on the replication of HSV-1 strain R-100 ($ACV^R$, $TK^A$) *

| | (-)-thalimonine | | | | | |
|---|---|---|---|---|---|---|
| **$Zn(asp)_2$** | 0 | $IC_{50}/8$ | $IC_{50}/4$ | $IC_{50}/2$ | $IC_{50}$ ♦ | $2IC_{50}$ |
| $2IC_{50}$ | 68 | 75 | 80 | 83.4 | 94 | 96.8 |
| $IC_{50}$ ° | 50 | 68 | 75 | 83.4 | 90 | 94 |
| $IC_{50}/2$ | 27 | 50 | 68 | 83.4 | 90 | 92 |
| $IC_{50}/4$ | 27 | 27 | 50 | 68 | 75 | 80 |
| $IC_{50}/8$ | 0 | 27 | 27 | 50 | 68 | 75 |
| 0 | 100 • | 0 | 0 | 27 | 50 | 68 |

* data shown in per cent inhibition as compared to the viral control; • viral control; infectious viral titre 4.33 $log_{10}$ i.u./0.1ml; ° 30 μM; ♦ 0.0000001 μM

Fig. 7 Combined effect of $Zn(asp)_2$ and (-)-thalimonine on the replication of HSV-1 strain R-100

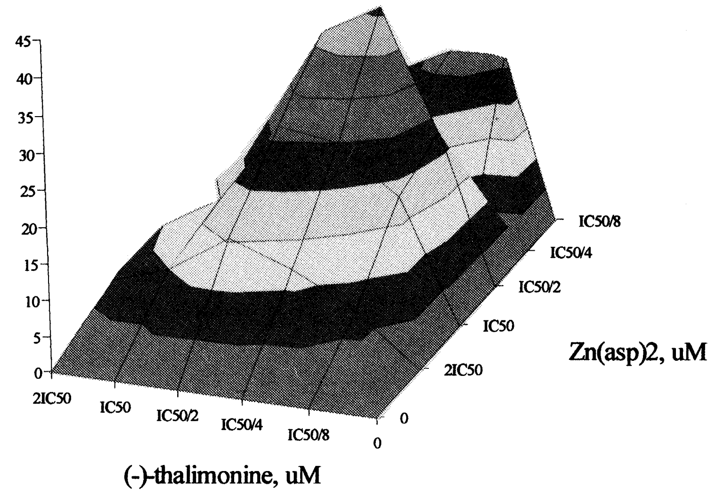

In summary, the data obtained demonstrate that the effect of dual combinations of zinc complexes and the pavine alkaloid (-)-thalimonine depends on the target cells specificity, i.e. when cells are uninfected the effect is antagonistic,while in HSV-1 infected cells the effect is synergistic.

**Acknowledgements:** This work was partially supported by the National Foundation of Science, Project L-810. We are thankful to Prof G. Palu from the Institute of Microbiology, University of Padova, Italy, for providing us with strain R-100. We are grateful to Prof. P. Bonchev from the Department of Analytical Chemistry, University of Sofia, for providing us with zinc complexes, and to Dr. M. Velcheva, Institute of Organic Chemistry with Centre of Phytochemistry, Bulgarian Academy of Sciences, for providing us with the alkaloid (-)-thalimonine.

**References**

1. T. L. Varadinova, P. R. Bonchev, C. K. Nachev, S. A. Shishkov, D. Strachilov, Z. Paskalev, A. Toutekova, M. Panteva, *J. Chemotherapy*, **5**, 3-9 (1993)
2. T. Varadinova, I. Pavlov, D. Dimitrov ###., *Compt. Rend. Acad. Bulg. Sci.*, **11**, 131-133 (1990)
3. T. L. Varadinova, S. A. Shishkov, N. D. Ivanovska, M.P.Velcheva, S. Danghaaghin, Z. Samadanghiin, Z. Yansanghiin, *Phytotherapy Res.*, **10**, 414-417 (1996)
4. J. S. Evans, K. P. Lock, B. A. Levine, J. N. Champness, M. R. Sanderson, W. C. Summers, P. J. McLeish, A. Buchan, *J Gen Virol*, **79**, 2083-2092 (1998)
5. C. Mengoli, F. Bevilacqua, G. Palu, *Current Topics in Mol. Pharmacol*, **1**, 33-48 (1993)
6. M. N. Prichard and C. Shipman, Jr, *Antiviral Res.*, **14**, 181-206 (1990)

